# Management of Fevers in Germany

**Published:** 1878-08-20

**Authors:** W. S. Caldwell

**Affiliations:** Vienna, Austria


### MANAGEMENT OF FEVERS IN GERMANY.

By W. S. Caldwell, M.D., Vienna, Austria.

In the following article I may not be able to advance any new ideas upon the subject of the management of the febrile state. I propose, however, to discuss certain methods of procedure which are regarded by most practitioners of medicine as either too impracticable for adoption in private practice, or as resting upon evidence in point of utility which is not quite trustworthy.

So much, in fact, has been written, of late, on the cold water treatment of typhoid fever, that the subject would seem to be well nigh exhausted; and the only apology that I have to offer for this encroachment upon an already fully occupied field, is that I hope that some of the suggestions I shall make will be more easily carried out in private practice than those usually given by men who have at their command the abundant resources of a well directed hospital.

Much, however, as this subject has been discussed, and in spite of the wonderful statistics lately published by Liebermeister, showing the beneficial effects of the cold bath as an antipyretic agent, during my late visits to the English and French hospitals I found that typhoid fever was *generally* treated by the administration of the mineral acids, much after the plan that I was taught in the the first lectures I attended a quarter of a century ago. In a course of lectures delivered by the professor of practical medicine in one of the New York colleges during the last winter, on the same subject, he never even referred to the benefits to be derived from the use of the cold bath in the management of this disease.

A late work on the nature and management of fevers (Ulber Wesen and Behandlung des Fiebers), by Dr. Carl Emil Buss, of Bale, is now attracting attention in Germany.

The writer is a warm advocate of the fermentation theory as a cause of the febrile state, and he says that the irritant is a living organism, which, by its multiplication in the circulation, calls forth the leison in each particular case.

"Others again claim that the presence of a living organism in the blood is not the *prime* cause of the complex symptoms, but that the products of decomposition which are produced by them, are more likely to act as an irritant, and thus cause the rise in temperature."

The injection of putrid matters into the circulation is found, he says, to produce septic fever in a direct ratio to the quantity of bacteria which such fluid contains. One of the most pernicious results that follow as a sequel of fevers, is a degeneration of the muscular substance of the heart, and a parenchymatous change in other organs essential to life. "Although hyperpyrexia may account in part for these results, it is probable that they are also largely produced by the presence of living animalculæ, which as yet we are unable to discern, but which act essentially the same as the trichina spiralis upon the muscles of the body when it has gained access to the system."

Taking this view of the nature of fevers in general, which the author claims are, in their manifestations, essentially the same, our remedies should be such as have a tendency to destroy these low forms of animal life. "It is wonderful th t those remedies which are the most powerful anti-pyretics, all possess in an eminent degree antiseptic properties also. In confirmation of the above theory, it is found that the remedies that act so happily in reducing animal heat when we have a high temperature, produce little or no effect when given to a healthy individual. It is very probable that the anti-pyretic properties of these drugs are effected by their combination within the circulation with the septic element that produced the fever, and in this way we have a favorable result produced."

While the author is a warm advocate of the use of cold baths to reduce the temperature in fevers, he thinks that their use should be supplemented by other treatment, for in such diseases as diphtheria, scarlatina and the worst forms of abdominal typhus, our patient is stricken down at once, and succumbs rapidly to the overpowering effects of a prime cause, that our symptomatic treatment fails to reach.

The indications in the treatment of fevers are, first, to neutralize the "causal irritant;" and, second, to use such other means as we have at our command, to assist in the reduction of the temperature.

To fulfill the first indication. the best remedies are the salicylates and cresotinic acid. Buss says, "the antipyretic effects of the salicylate of soda cannot be equalled by any other drug. It is far preferable to the salts of cinchona, because it does not retard the elimination of carbonic acid in the system, and acts more energetically in reducing the animal heat. Within three or four hours after the drug was given, the fall of temperature was generally from two to three degrees, but when given largely, the reduction was as much as five degrees. The formula for the use of this remedy in typhoid fever is as follows:

| | |
|---|---|
| Salicylate of soda.................... | 15 |
| Syrup of cinnamon.................... | 30 |
| Water.................................... | 180 Mix. |

Give one tablespoonful every three hours, excepting eight hours of the night, when the patient is allowed to sleep.

Should this dose offend the stomach, he advises to give one-third the quantity and repeat the dose every hour.

Cresotinic acid, given to the extent of 10 to 15 grams in the 24 hours, was found equally efficacious.

The effect of these remedies was, as the author asserts, to shorten the duration of typhoid fever. Their internal use was in most cases supplemented by the use of the cold bath, but as this part of the subject is not very different from that given by Liebermeister, I will not quote the latter in this connection.—*Journal and Examiner.*

